# Complete Remission of Human Parvovirus B19 Associated Symptoms by Loxoprofen in Patients with Atopic Predispositions

**DOI:** 10.1155/2012/703281

**Published:** 2012-04-24

**Authors:** Itsuro Kazama, Naoko Sasagawa, Toshiyuki Nakajima

**Affiliations:** ^1^Department of Physiology I, Tohoku University Graduate School of Medicine, Seiryo-cho, Aoba-ku, Sendai, Miyagi 980-8575, Japan; ^2^Department of Internal Medicine, Iwakiri Hospital, Miyagino-ku, Sendai, Miyagi 983-0821, Japan

## Abstract

Two cases of women in their thirties with past histories of atopic dermatitis and allergic rhinitis developed a low grade fever, followed by a butterfly-shaped erythema, swelling of their fingers, and polyarthralgia. Despite such symptoms that overlap with those of systemic lupus erythematosus (SLE), the diagnostic criteria for SLE were not fulfilled. Due to positive results for human parvovirus B19 (HPV-B19) IgM antibodies in the serum, diagnoses of HPV-B19 infection were made in both cases. Although acetaminophen failed to improve their deteriorating symptoms, a nonsteroidal anti-inflammatory drug (NSAID), loxoprofen, completely removed the symptoms immediately after the administration. In those cases, since the patients were predisposed to atopic disorders, an increased immunological response based on the lymphocyte hypersensitivity was likely to be involved in the pathogenesis. The immunomodulatory property of NSAID was thought to repress such lymphocyte activity and thus provided a rapid and sustained remission of the disease.

## 1. Introduction

The classic symptoms of human parvovirus B19 (HPV-B19) infection include fever, rash, arthralgia, and edema [1]. In children, the symptoms are usually self-limited, resolving spontaneously within a few weeks [2]. In adults, however, they are often prolonged, sometimes causing serious complications, such as chronic arthritis, myocarditis, and meningitis [3–5], or triggering the onset of autoimmune diseases [6]. According to previous studies, the direct cytotoxicity of the virus has been ascribed to the pathogenesis of the symptoms [7, 8]. However, due to the absence of drugs that are effective for the virus, there is no causal treatment. To relieve the symptoms, analgesics or antipyretics are generally used [9], although they do not shorten the duration of the disease. Here, we experienced two cases of HPV-B19 infection in adult patients with past histories of atopic dermatitis and allergic rhinitis. In those cases, due to the atopic predispositions of the patients, an increased immunological response was more likely to be involved in the pathogenesis of the symptoms. In such cases, a nonsteroidal anti-inflammatory drug (NSAID), loxoprofen, dramatically improved their deteriorating symptoms immediately after the administration. The immunomodulatory property of this drug was thought to repress the lymphocyte activity, and thus provided a rapid and sustained remission of the disease.

## 2. Case Presentation

### 2.1. Case 1

**Figure 1:**
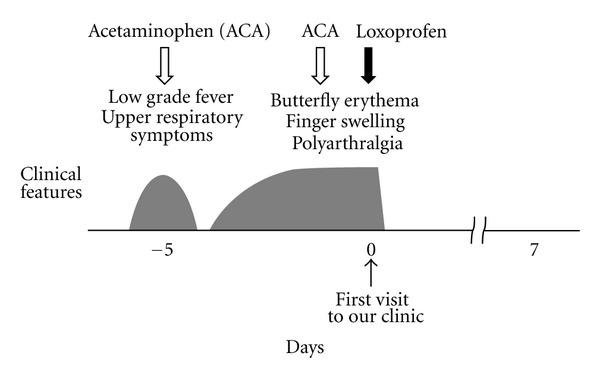
Clinical course of *Case  1*. Five days prior to her visit, the patient had upper respiratory symptoms and a low grade fever. Then, she developed a butterfly-shaped erythema, swelling of her fingers, and polyarthralgia. Immediately after the initiation of loxoprofen, the symptoms rapidly disappeared within 12 hours. No recurrence of the symptoms or signs was noted afterwards. ACA, acetaminophen.

A 38-year-old woman with a childhood history of atopic dermatitis and allergic rhinitis came to our outpatient clinic because of polyarthralgia and swelling of her fingers. Five days prior to her visit, she had upper respiratory symptoms and a low grade fever (Figure 1). Oral administration of 400 mg acetaminophen relieved those symptoms. Then, however, she developed a butterfly-shaped erythema on her face and swelling of her fingers. Three days later, although the facial erythema had improved, she developed joint pain in her fingers, wrists, shoulders, and knees, for which acetaminophen did not work. On physical examination, the patient looked tired. Her body temperature was 36.7°C, blood pressure was 94/50 mmHg, and pulse rate was 61 beats/min. She weighed 48 kg and was 153 cm tall. She had no rash except for a malar erythema that was faint at that time. All her fingers were swollen, but her wrist, shoulder, and knee joints were not. Both her legs had pretibial pitting edema. Laboratory data showed mild hypoproteinemia (serum protein 6.4 g/dL) and slightly decreased peripheral white blood cell count (4,100/*μ*L). However, other hematologic parameters were relatively normal (hemoglobin 11.2 g/dL, hematocrit 35.2%, reticulocyte 1.4%, and platelet count 230,000/*μ*L). Liver enzymes and C-reactive protein levels were not elevated. Regardless of hypocomplementemia (C3 72 mg/dL, C4 9 mg/dL, and CH50 18.6 U/mL), the tests for serum antinuclear antibodies and rheumatoid factor were negative. Althoughthe IgG antibody was not examined, a positive result for HPV-B19 IgM antibody (index 7.97) indicated a recent infection of the virus [10]. Since microbiological tests for other viruses, such as measles and rubella, were negative, a diagnosis of HPV-B19 infection was finally made. Because her symptoms deteriorated despite the use of acetaminophen, oral administration of loxoprofen (180 mg/day) was alternatively started immediately after the diagnosis (Figure 1). The symptoms, including facial erythema, polyarthralgia, and the swelling of her fingers, rapidly disappeared within 12 hours after the initiation of the drug. No recurrence of the symptoms or signs was noted afterwards, indicating the complete remission of the disease.

### 2.2. Case 2

**Figure 2:**
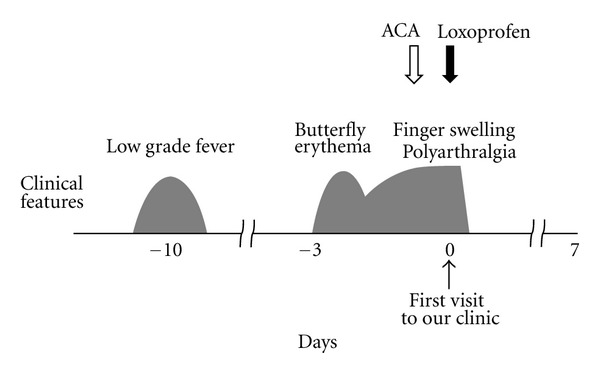
Clinical course of *Case  2*. Ten days prior to her visit, the patient had a low grade fever. Seven days later, she developed a butterfly-shaped erythema, followed by swelling of her fingers and polyarthralgia. Immediately after the initiation of loxoprofen, the symptoms quickly disappeared within 12 hours. There was no recurrence of the symptoms or signs afterwards. ACA, acetaminophen.

A 36-year-old woman with a past history of allergic rhinitis came to our outpatient clinic because of polyarthralgia and swelling of her fingers. Ten days prior to her visit, she had a low grade fever (Figure 2) when her 9-year-old son was diagnosed as having erythema infectiosum because of his slapped cheek rash. Seven days later, she developed a butterfly-shaped erythema transiently on her face that disappeared the next day. Then, she developed swelling of her fingers and joint pain in her shoulders, ankles, and knees. Since oral administration of acetaminophen did not relieve her symptoms, she came to our clinic. On physical examination, the patient looked tired. Her body temperature was 37.0°C, blood pressure was 110/72 mmHg, and pulse rate was 66 beats/min. She weighed 52 kg and was 160 cm tall. At that time, she presented no malar erythema and no rash in her body. Although all her fingers were swollen, the swelling of her shoulder, ankle, and knee joints was not obvious. Laboratory data showed normal hematologic parameters (white blood cell count 6,300/*μ*L, hemoglobin 12.6 g/dL, hematocrit 37.6%, and platelet count 264,000/*μ*L). Liver enzymes and C-reactive protein levels were not elevated. Due to a positive result for HPV-B19 IgM antibody in the serum (index 8.20) and the preceding infection of her son, a diagnosis of HPV-B19 infection was made. Oral administration of loxoprofen (180 mg/day) was immediately started after the diagnosis (Figure 2). Her deteriorating symptoms, including polyarthralgia and the swelling of her fingers, quickly disappeared within 12 hours after the initiation of the drug.

## 3. Discussion

Skin rashes and arthralgia are the most common clinical features in adult patients with HPV-B19 infection [1]. Previous studies have ascribed the pathogenesis of the symptoms to the direct cytotoxicity of the virus [7, 8]. In our cases, however, it was less likely involved, because both patients developed the symptoms in the absence of reticulocytopenia or thrombocytopenia that usually coincides with viremia [11, 12]. Although their clinical and laboratory features did not fulfill the diagnostic criteria for systemic lupus erythematosus (SLE) [13], their SLE-like features, including butterfly-shaped erythema and polyarthralgia, strongly suggested an increased immunological response. In addition, hypocomplementemia in *Case  1*, which occurs in both SLE and HPV-B19 infection [14], indicated the activation of the classical complement pathway caused by the increase in immune complexes. Therefore, in our cases, such enhanced immunity was more likely to be involved in the pathogenesis of the symptoms. According to previous studies, patients with atopic dermatitis and allergic rhinitis are prone to develop autoimmune diseases due to the hypersensitivity of T-helper 2 lymphocytes [15, 16] or the dysregulated activity of B-lymphocytes [17]. In our cases, since both patients were predisposed to atopic disorders, such as atopic dermatitis and allergic rhinitis, the HPV-B19 infection may have triggered the activity of the lymphocytes.

For adult patients with HPV-B19 infection, NSAIDs are often used to provide symptomatic relief for arthralgia and myalgia [12, 18]. However, since these drugs do not shorten the duration of the disease, the patients have to wait at least 1 to 2 weeks for the spontaneous remission of the disease [19, 20]. In our cases, although acetaminophen failed to ameliorate the patients' symptoms, loxoprofen completely removed their deteriorating symptoms quickly after the administration (Figures 1 and 2). Therefore, in our cases, loxoprofen was thought to be responsible for the complete remission of the disease. NSAIDs are the most commonly used anti-inflammatory, analgesic, and antipyretic drugs. Additionally, due to their immunomodulatory properties [21], they have also been used in the treatment of autoimmune diseases, such as SLE [22] and rheumatoid arthritis [23]. Studies have revealed that NSAIDs inhibit the migration of leukocytes or their cytokine production, either cyclooxygenase (COX) dependently [24] or independently [25, 26], and thus exert immunomodulatory effects. In the present cases, since the increased activity of lymphocytes was mainly involved in the pathogenesis, the immunomodulation by the NSAID was thought to be responsible for the rapid removal of the symptoms and the complete remission of the disease. Recently, we have demonstrated in a basic study that NSAIDs repress the activity of lymphocytes by the functional inhibition of delayed rectifier K^+^-channels (Kv1.3) [27]. Since T-lymphocytes highly express the channels in their plasma membranes [28], such mechanism may have greatly contributed to the immunomodulatory effect of NSAID in our patients. In this regard, besides the short-term use of immunosuppressive agents or steroids, the use of selective Kv1.3-channel blockers may also be useful. The early administration of such drugs would not only shorten the duration of the disease, but also prevent the serious complications caused by HPV-B19 infection or the onset of autoimmune diseases triggered by the virus.

In summary, this is the first report of HPV-B19 infection in adult patients with atopic predispositions showing the usefulness of NSAID for the quick removal of the symptoms. The immunomodulatory property of NSAID was thought to repress the increased lymphocyte activity and thus provided a rapid and sustained remission of the disease.

## References

[1] Woolf AD, Campion GV, Chishick A (1989). Clinical manifestations of human parvovirus B19 in adults. *Archives of Internal Medicine*.

[2] Lindblom A, Isa A, Norbeck O (2005). Slow clearance of human parvovirus B19 viremia following acute infection. *Clinical Infectious Diseases*.

[3] Finkel TH, Torok TJ, Ferguson PJ (1994). Chronic parvovirus B19 infection and systemic necrotising vasculitis: opportunistic infection or aetiological agent?. *The Lancet*.

[4] Heegaard ED, Eiskjær H, Baandrup U, Hornsleth A (1998). Parvovirus B19 infection associated with myocarditis following adult cardiac transplantation. *Scandinavian Journal of Infectious Diseases*.

[5] Douvoyiannis M, Litman N, Goldman DL (2009). Neurologic manifestations associated with parvovirus B19 infection. *Clinical Infectious Diseases*.

[6] Meyer O (2003). Parvovirus B19 and autoimmune diseases. *Joint Bone Spine*.

[7] Schwarz TF, Wiersbitzky S, Pambor M (1994). Case report: detection of parvovirus B19 in a skin biopsy of a patient with erythema infectiosum. *Journal of Medical Virology*.

[8] Nikkari S, Roivainen A, Hannonen P, Mottonen T, Luukkainen R, Yli-Jama T (1995). Persistence of parvovirus B19 in synovial fluid and bone marrow. *Annals of the Rheumatic Diseases*.

[9] Naides SJ, Scharosch LL, Foto F, Howard EJ (1990). Rheumatologic manifestations of human parvovirus B19 infection in adults: Initial two-year clinical experience. *Arthritis and Rheumatism*.

[10] Young NS, Brown KE (2004). Parvovirus B19. *The New England Journal of Medicine*.

[11] Anderson MJ, Higgins PG, Davis LR (1985). Experimental parvoviral infection in humans. *Journal of Infectious Diseases*.

[12] Heegaard ED, Brown KE (2002). Human parvovirus B19. *Clinical Microbiology Reviews*.

[13] Liang MH, Fortin P, Schneider M (2004). The American College of Rheumatology response criteria for systemic lupus erythematosus clinical trials: measures of overall disease activity. *Arthritis and Rheumatism*.

[14] Nesher G, Osborn TG, Moore TL (1995). Parvovirus infection mimicking systemic lupus erythematosus. *Seminars in Arthritis and Rheumatism*.

[15] Feizy V, Ghobadi A (2006). Atopic dermatitis and systemic autoimmune diseases: a descriptive cross-sectional study. *Dermatology Online Journal*.

[16] Takeoka K, Hidaka Y, Hanada H (2003). Increase in serum levels of autoantibodies after attack of seasonal allergic rhinitis in patients with graves’ disease. *International Archives of Allergy and Immunology*.

[17] Astrakhan A, Omori M, Nguyen T (2007). Local increase in thymic stromal lymphopoietin induces systemic alterations in B cell development. *Nature Immunology*.

[18] Broliden K, Tolfvenstam T, Norbeck O (2006). Clinical aspects of parvovirus B19 infection. *Journal of Internal Medicine*.

[19] Yoshidome Y, Hayashi S, Okadome T, Maruyama Y, Nishitarumizu K (2004). Eight cases of human parvovirus B19 arthropathy. *Nihon Naika Gakkai Zasshi*.

[20] Ichinose K, Migita K, Sekita T (2004). Parvovirus B19 infection and myofasciitis. *Clinical Rheumatology*.

[21] Cho JY (2007). Immunomodulatory effect of nonsteroidal anti-inflammatory drugs (NSAIDs) at the clinically available doses. *Archives of Pharmacal Research*.

[22] Wallace DJ (2010). Advances in drug therapy for systemic lupus erythematosus. *BMC Medicine*.

[23] Gotzsche PC (1989). Methodology and overt and hidden bias in reports of 196 double-blind trials of nonsteroidal antiinflammatory drugs in rheumatoid arthritis. *Controlled Clinical Trials*.

[24] Iniguez MA, Punzon C, Fresno M (1999). Induction of cyclooxygenase-2 on activated T lymphocytes: regulation of T cell activation by cyclooxygenase-2 inhibitors. *Journal of Immunology*.

[25] Hackstein H, Morelli AE, Larregina AT (2001). Aspirin inhibits in vitro maturation and in vivo immunostimulatory function of murine myeloid dendritic cells. *Journal of Immunology*.

[26] Gao JX, Issekutz AC (1993). The effect of ebselen on polymorphonuclear leukocyte migration to joints in rats with adjuvant arthritis. *International Journal of Immunopharmacology*.

[27] Kazama I, Maruyama Y, Murata Y Suppressive effects of nonsteroidal anti-inflammatory drugs diclofenac sodium, salicylate and indomethacin on delayed rectifier K^+^-channelcurrents in murine thymocytes.

[28] Lewis RS, Cahalan MD (1995). Potassium and calcium channels in lymphocytes. *Annual Review of Immunology*.

